# Dr. L. H. Kitchel

**Published:** 1903-07

**Authors:** 


Dr. L. H. Kitchel of Alden, N. Y. died April 20, 1903, aged 57 years. He graduated at the University of Buffalo in 1870.

